# Minutes of the Fourth Annual Meeting of the American Medical Association, Held in the City of Charleston, May 1, 1851

**Published:** 1851-07

**Authors:** 


### Part 3.—Selections.

ARTICLE I.

*Minutes of the Fourth Annual Meeting of the American Medical Association, held in the City of Charleston, May 1, 1851.*

CHARLESTON, May 6th, 1851.

The Association met in St. Andrew's Hall at 11 o'clock A. M., the President, Dr. Mussey, in the chair.

Dr Simons, chairman of a committee from the South Carolinia Medical Association, welcomed the delegates to the city.

Dr. Frost, chairman of the Committee of Arrangements, read a list of the delegates who had registered their names.

The President read the following letter:

"PHILADELPHIA, Sept. 27th, 1850.

"R. D. MUSSEY, M.D., President of the American Medical Association.

SIR: The state of my health is such as to render my attention to professional duties, for the present, unsafe, and I have, therefore, resolved to seek relaxation abroad. As I shall not, in all probability, return home before the next meeting of the Association, I beg leave to tender my resignation of the office of Secretary, and to offer to the Association, through you, my acknowledgments for the honor with which they have so repeatedly distinguished me.

"With most earnest hopes for the prosperity of the Association, I have the honor to be

"Very respectfully, your ob't serv't,

[Signed] "ALFRED STILLE."

On motion of Dr. Arnold, of Ga., the letter was ordered to be recorded on the minutes, in evidence of the high estimation felt by the Association for the services of Dr. Stille.

Dr. Arnold, of Ga., offered the following resolution, which was seconded by Dr. Frost, of S. C., and adopted.—

Resolved, That a committee of one from each State represented in the Association, to be chosen by their respective delegations, be appointed to nominate suitable officers to be elected for the ensuing year.

On motion, the association then went into a recess of fifteen minutes, to enable the delegations to appoint the Nominating Committee.

At the expiration of the recess, the President annouuced the Nominating Committee as follows: Drs. Horatio Adams, Massachusetts; Usher Parsons, Rhode Island; A. H. Stevens, New York; Joseph Fithian, New Jersey; Joseph Carson, Pennsylvania; G. W. Miltenberger, Maryland; B. R. Welford, Virginia; N. J. Pittman, North Carolina; H. R. Frost, South Carolina; R. D. Arnold, Georgia; W. H. Anderson, Alabama; James Jones, Louisiana; J. B. Lindsley, Tennessee; J. B. Flint, Kentucky; Geo. Mendenhall, Ohio; Thos. Reyburn, Missouri; John Sloan, Indiana; C. Boyle, Dist of Columbia.

The President delivered an address.

The Nominating Committee reported the following officers of the Association.

President—James Moultrie, South Carolina.

Vice-Presidents—G. Hayward, Mass.; R. D. Arnold, Ga.; B. R. Welford, Va.; J. B. Flint, Ken.

Secretaries—H. W. De Saussure, S. C.; P. C. Gooch, Va.

Treasurer—Isaac Hays, Pennsylvania.

The report was accepted, and on motion of Dr. La Roche, of Pennsylvania, it was resolved that the officers thus nominated be the officers of the Association the year; and that the officers elected be invited to take their seats. The President elect then returned his thanks to the Association, for the honor conferred upon him.

The Secretary read a report transmitted to him by Dr. Roberts, of Maryland, of a committee appointed at the last meeting of the Assoctation to report unfinished business. On motion of Dr. Arnold, of Ga., the report was accepted and laid on the table.

Dr. Gaillard, of S. Carolina, moved that the following resolution, offered by Dr. Drake, of Ohio, at the session of 1850, be taken up for consideration, viz:—

Resolved, That the second section of the regulations of the Association be so amended as to require that candidates for membership by invitaton be nominated in writing by five members; that when elected, they shall enjoy all the rights of delegates; and that all permanent members shall be entitled to vote.

After some discussion, on motion of Dr. Stevens, of New York, the resolction was referred to a committee consisting of

Dr. Drake, of Ohio, Dr. Wood, of Pennsylvania, and Dr. Wellford, of Virginia.

The reports of standing committees being then called for, Dr. Stevens, of New York, offered the following resolution:

Resolved, That a committeee be appointed to report to the Association the business before it, and to offer such suggestions as they may deem advisable for the due discharge of the same.

The resolution was discussed by Drs. Storer, of Mass.; Moore, of Ga.; and Bellinger, of S. C., and finally rejected.

On motion, the Association adjourned to meet on Wednesday, the 7th, at 10 o'clock A. M.

MAY 7TH.—*Morning Session.*

The Association met at 11 o'clock A. M., the President, Dr. Moultrie, in the chair.

Dr. Wood, of Penn., chairman of the committee appointed to consider and report on the amendment to the constitution, proposed in the resolution of Dr. Drake, of Ohio, at the session of '50, reported that it was advisable to adopt that part of the resolution requiring members by invitation to be proposed, in writing, by five members, giving them all the privileges of delegates, except the right to vote; also recommending that the part of the resolution giving permanent members the right to vote, be adopted.

Dr. Drake, of Ohio, from the Same committee, made a minority report, recommending that the constitution be so amended as to require members by invitation to be nominated in writing by five members, giving them all the rights and privileges of delegates, including those of voting and permanent membership; also recommending that permanent members be allowed the privilege of voting. These reports were discussed by Drs. Arnold, of Ga.; Wood, of Penn.; Davis, of Ill.; Drake, of Ohio; Atlee, of Penn.; Meigs, of Penn.

At this stage of the discussion, Dr. C. Hooker, of Conn., asked and obtained a suspension of the discussion, to enable him to offer the following resolution, which was adopted;

Resolved, that no member be permitted to speak longer than ten minutes at one time, in any one debate.

The discussion of the report was then resumed by Drs. Dickson, of S. C.; Hays, of Penn.; Davis, of Ill.; Bond, of Md.; and Z. B. Adams, of Mass.

Page(s) missing

Dr. Hays, of Penn., moved to lay the reports on the table, but modified the motion so that a vote might be taken on each recommendation of the reports. The part of the report requiring members by invitation to be nominated, in writing, by five members, and giving them the rights of delegates, was then lain on the table. The vote being taken on allowing permanent members the privilege of voting, it was lost by a large majority.

Dr. Hays, of Penn., Treasurer and Chairman of the Committtee on Publication, presented and read his reports, concluding with the following resolutions:

Resolved, That the assessment for the present year shall be three dollars.

Resolved, That those delegates who pay the assessment shall be entitled to one copy of the Transactions for the present year, and that the payment of two dollars in addition shall entitle them to two additional copies.

Resolved, That the permanent members shall be entiled to one copy of the Transactions for the present year, on the payment of two dollars, and three copies on the payment of five dollars.

Resolved, That the societies which have been represented in the Association shall be entitled to copies, for their members, on the same terms that copies are furnished to permanent members.

Resolved, That permanent members, unless present at the meeting as delegates, shall not be subject to any assessment.

Resolved, That the several committees be requested to bring to the meeting of the Association their reports correctly and legibly transcribed, and that they be required to hand them to the Secretaries as soon as they have been read.

The report was accepted, and referred to the Committee on Publication.

The resolutions were considered *seriatim*, and adopted.

On motion of Dr. Drake, of Ohio, the report of the Committee on Surgery was called for.

Dr. Paul F. Eve, of Ga., chairman of the Committee on Surgery, presented and read the report of the committee, which, on motion, was accepted and referred to the Committee on Publication.

The Secretary informed the Association that he had received from Dr. Flint, of New-York, chairman of the Committee on Practical Medicine, a printed copy of the report of the committee, three hundred of which had also been sent for distribution among the members of the Association.

On motion of Dr. Hays, of Penn., the report was read by its title, and referred to the Committee on Publication.

The Association then adjourned to 5 P. M.

*Afternoon Session.*

The President in the chair.

Dr. Boyle, of the Dist. of Columbia, invited the Association, in the name of the Medical Society of the District of Columbia, to hold its next annual meeting in the city of Washington. Drs. C. P. Johnson and P. C. Gooch, of Va., presented similar invitations from the Medical Department of Hampden Sydney Ccllege and the Medical Society of Virginia, to meet in Richmond, Va. Dr. James Jones, of Louisiana, presented an invitation of like purport from the Medical Faculty of the University of Louisiana, to hold its next session in New-Orleans, La.

Dr. Reyburn, of Missouri, urged the claims of St. Louis as the next place of meeting.

On motion, these several invitations were referred to the Committee on nominations.

On motion of Dr. Parsons, of Rhode Island, the Committee on Nominations were requested to make the appointment of the several standing committees. Drs. N. B. Ives, of Conn., and N. S. Davis, of Ill's, were added to the Committee on Nominations, these States not having been previously represented. Dr. Jones, of Louisiana, declined serving on the committee, and Dr. E. D. Fenner was appointed, by the delegation of the State, in his stead.

On motion of Dr. Wragg, of S. C., the reports of the Committee on Prize Essays, and of Obstetrics, were made the special order of the evening session.

The Secretary presented and read the report of the Committee on Prize Essays. The committee awarded the prize for successful authorship to Dr. John C. Dalton, Jr., of Boston, author of the essay "On the Corpus Luteum of Menstruation and Pregnancy," recommending that the essay be published in the Transactions of the Association. On motion, the report was accepted and referred to the Committee on Publication.

Dr. Storer, of Massachusetts, the chairman of the Committee on Obstetrics, presented and read the report of the committee. Dr. Robinson, of S. C., moved the report to be re-committed to the committee. After some discussion, this resolution was adopted. The report was recommitted and made the special order for the morning session.

The Association then adjourned to 10 A. M., of Thursday.

May 8th—*Morning Session.*

The President, Dr. Moultrie, in the chair.

The minutes of the last meeting were read and confirmed.

Dr. J. N. Smith, of Mass., moved that the report of the committee on Medical Education be made the special order, after the disposal of the report of the Committee on Obstetrics.

Dr. Gaillard, of S. C., on behalf of the Committee of Arrangements, read a list of the delegates registered since the last report.

Dr. Campbell, of Georgia, placed on the table, for inspection and examination, a model of a malformation of the knee-join, the patella of which was wanting.

Dr. G. B. Wood, of Pa., offered the following resolution:—

Resolved, That colleges, exclusively of dentistry, and pharmacy, are not recognized by this Association as among the bodies authorized to send delegates to its meetings.

Dr. J. R. Wood, of New-York, moved to amend by dividing the resolution so as to take the question—first, on the reception of delegates from colleges of dentistry, and then on the reception of delegates from colleges of pharmacy. The amendment was accepted.

The question of the reception of delegates from colleges of dentistry was then discussed by Drs. Hays, of Pa.; Z. B. Adams, of Mass.; Wood, of Penn.; Drake, of Ohio; Wood, of N. York; J. L. Atlee, of Penn.; and Bond, of Maryland.

At this stage of the discussion, Dr. Yardley, of Penn., asked and obtained leave to read the following preamble and resolutions, ordered to be laid before the Association by the Philadelphia County Medical Society.

Whereas, The Constitution of the American Medical Association, by providing for the reception of delegates from all permanently organized medical societies, medical colleges, hospitals, lunatic asylums, and other permanently organized medical institutions, unjustly favors the profession in cities where such institutions exist, and can be readily formed, and diminishes the importance and thereby discourages the formation of county medical societies in rural districts, therefore,

Resolved, That the Constitution of said Association should be altered so as to admit only delegates from county or state medical societies.

Resolved, That a copy of the preceding preamble and resolution, signed by the President and Secretary of this Society, be transmitted to the Association at its next session.

[Signed.] SAMUEL JACKSON, Pres't,
D. FRANCIS CONDIE, Sec'y.

PHILADELPHIA, April 25th, 1851.

The preamble and resolution having been read, Dr. Lamb, of Penn., moved to lay the whole subject on the table, which motion was lost. The discussion was then resumed by Drs. Hays, of Penn., Atlee, of Penn., Drake, of Ohio, Wood, of Penn., and Bond, of Maryland.

Dr. J. L. Atlee, of Penn, moved that the whole subject of delegates from colleges of dentistry and pharmacy be referred to a committee consisting of five members, with instructions to report whether some more equitable plan of representation than the present one cannot be devised. This resolution was adopted. On motion of Dr. Yardley, of Penn., the preamble and resolutions of the Philadelphia County Medical Society were referred to the same committee.

Dr. Jones, of N. C., offered the following resolution:

Resolved, That all the Medical Colleges in the U. States are hereby earnestly and respectfully requested to hold a convention through delegates respectively chosen by them, a least once in every six years, to take into consideration the proper method of harmoniously elevating the standard of medical education in said colleges.

The Secretary read a letter and some documents placed on the table, which, on motion of Dr. C. Hooker, of Conn., were referred to the Indiana delegation, with instructions to report at 1 o'clock P. M.

The report of the Committee on obstetrics being the special order, Dr. Storer, of Mass., chairman of that committe, presented the report, which on motion was accepted and referred to the Committee on Publication.

Dr. J. L. Atlee, of Penn., presented to the Association, on behalf of Prof. Haldeman, of Lancaster County, Penn., an essay on Latin pronunciation, and moved that it be referred to the Committee on Medical Education.

The report of the Committee on Medical Education being now the special order, its reading was suspended to receive the report of the Nominating Committee, which was presented, read, and laid on the table.

Dr. Hays, of Penn., called up a resolution submitted by himself during the session of 1849, which, as it involved an alteration of the constitution, was laid on the table until the next session. In consequence of the absence of Dr. Hays from the session of 1850, the resolution was referred to the committee on unfinished business, to be reported on at the present session. The resolution reads as follows: (vide vol. ii. pp. 43.)

Resolved, To strike out from the Constitution of the Association all that relates to standing committees on "Medical Sciences," on "Practical Medicine," on "Surgery," on "Obstetrics," on "Medical Education," and on "Medical Literature.

This resolution was discussed by Drs. Stevens, of N. York, Drake, of Ohio, Hooker, of Conn., Wood, of Penn., and Davis, of Ill., and finally adopted.

Dr. Wood, of Penn., offered the following resolution, which was adopted:

Resolved, That a committee of seven be appointed to take into consideration the arrangement of committees for future action, to report as early as possible.

The roport of the Indiana delegation, being the special order for one o'clock P. M., was presented and read. The report recommended that the letter and accompanying documents be referred to the Medical Society of the State of Indiana, for their consideration. On motion the report was accepted, and the documents directed to be sent to the Medical Society of of the State of Indiana.

The report of the committee on Medical Education being now the special order, Dr. Johnson, of Missouri, moved that, as the report was a long one, and the hour late, the reading of the report be made the special order for the evening session, and the report of the Committee on Medical Literature be called up in its stead. The motion was adopted.

Dr. Reyburn, of Missouri, chairman of the Committee on

Medical Literature, presented and read the report of the committee. In the course of his report, he gave way to a motion to adjourn.

Before the adjournment, the Secretary announced that Dr. N. S. Davis, of Illinois, had prepared a paper entitled, "An Experimental Inquiry concerning some points connected with the Process of Assimilation and Nutrition."

On motion of Dr. Stephenson, of New-York, Dr. Davis was requested to read his paper after the reports of the standing committees had been read and disposed of.

The Association then Adjourned to five o'clock P. M.

*Afternoon Session.*

The President, Dr. Moultrie, in the chair.

The President announced that he had appointed Drs. Hays, of Penn., Stevens, of N. Y., Yardley, of Penn, Storer, of Mass., and Jones, of N. C., the committee under Dr. Wood's resolution, to inquire into the constitutionality of receiving delegates from colleges of dentistry and pharmacy; also that he had appointed Drs. G. B. Wood, of Penn., Drake, of Ohio, Stevens, of N. Y., Hooker, of Conn., Adams, of Mass., B. R. Wellford, of Va., and Dickson, of S. C., the committee under Dr. Wood's resolution to appoint special committees in place of the standing committees abolished by the adoption of Dr. Hays' resolution.

Dr. Dickson, of S. C., asked and obtained leave, before proceeding with the special order, to introduce the following preamble and resolutions:—

Whereas, efforts are being made to repeal the law of 1847, which confers protective rank on the members of the Medical Department of the Army: Therefore,

Resolved, That the American Medical Association views with regret the existence of hostility to the act of Congress approved February 11th, 1847, which confers legal rights, and equality with other staff departments, on the Medical Officers of the Army, and gives them a position to which the character and importance of the profession entiles them.

Resolved, That copies of these resolutions, with the resolutions of the Association passed at its last annual meeting, on the same subject, be transmitted to the Secretary of War, and of the Navy, also to the Chiefs of the Medical Department of of each service, and to the presiding officer of the Senate and House of Representatives of the United States.

The resolutions were seconded by Dr. Lebby, of S. C., and unanimously adopted.

Dr. Reyburn, chairman of the Committee on Medical Literature, completed the reading of the report of the committee, and concluded with the following resolution:—

Resolved, That a special committee be appointed to take into consideration that portion of this report which refers to the organization of a society for the reception and discussion of original scientific papers, and that said committee be and is hereby instructed to present the details of a plan, if deemed by them expedient, for such a society or section at the next annual meeting.

On motion of Dr. Lamb, of Penn, the report was accepted and referred to the Committee on Publication, and the resolution was referred to the Committee on the Organization of the Association, appointed under Dr. Wood's resolution.

The report of of the Committee on Medical Education being now the special order, it was moved that that part of the report relating to demonstrative midwifery, referred to the committee by resolution of the Association at its last meeting, be now read, and the remainder of the report be made the special order of the morning session, The motion was adopted.

Dr. Hooker, of Conn., chairman of the Committee on Medical Education, then presented and read the report ot the committee on demonstrative midwifery. On motion the report was accepted and referred to the Committee on Publication.

Dr, Dickson, of S. C., then offered the following resolution, which was unanimously adopted:

Resolved, That this Association unanimously approve the opinion expressed in the report of the committee on Medical Education in respect to demonstrative midwifery.

The Association then adjourned to ten o'clock A. M. of Friday.

MAY 9TH—*Morning Session.*

The President, Dr. Moultrie in the chair.

The minutes of the last meeting were read and confirmed.

Dr. Stevens, of N. Y., asked and obtained a suspension of the special order, which was the report of the Committee on Medical Education, to enable him to present the following resolutions:

Resolved, That the members of this Association cannot separate without expressing their grateful sense of the hospitalities and numerous delicate attentions received from their medical brethren of South Carolina, and the citizens of Charleston.

Resolved, That a committee of three be formed to procure a tablet, with a suitable inscription commemorative of this meeting, and of the feelings it has elicited, to be placed at the disposal of the Medical Association of the State of South Carolina.

The inscription on the label to be as follows:—

"This tablet is here placed by the American Medical Association, to commemorate their annual meeting in the city of Charleston, in May, 1851, and to signalize their gratitude for the extraordinary professional and social enjoyment that accompanied it."

The resolutions were seconded by F. A. Ramsey, of Tenn., and adopted. Drs. G. Hayward, of Mass.; Stevens, of N. Y.; and Ramsey, of Tenn., were appointed the committee.

Dr. Ramsey, of Tenn., asked and obtained a further suspension of the rules, to read a letter from Dr. E. D. Fenner, in relation to his annual publication of a volume of "Reports on the Diseases of the Southern States." The letter was read and Dr. Ramsey, of Tenn., offered the following resolution, which was adopted:

Resolved, That the effort of Dr. Fenner to place, on a firm and durable basis, an annual publication embracing medical reports from the whole Southern portion of the Union, merits the commendation of this Association, and should receive solid support from American physicians.

Dr. Hays, of Penn., asked and obtained leave to call up so much of the report of the nominating committee as recommends a place for the next meeting of the Association, and nominates the Committees of Arrangement and Publication. The report was read as follows:

The Committee of Nominations beg leave to report that they recommend, as the place for the next meeting of the Association, Richmond, Va.

They report, also, the following committees for the confirmation of the meeting:

*Committee of Arrangements.*—Drs. R. W. Haxall, Va., ch'n; C. P. Johnson, Va.; C. B. Gibson, Va.; J. Beale, Va.; C. S. Mills, Va.; S. Mupas, Va.; R. D. Haskins, Va.; M. P. Scott, Va.

*Committee on Publication.*—Drs. Isaac Hays, of Penn, ch'n; G. Emerson, Penn., D. F. Condie, Penn.; H. W. De Saussure, S. C.; Isaac Parrish, Penn.; G. W. Norris, Penn.; P. C. Gooch, Virginia.

Dr. Drake, of Ohio, made an urgent appeal in favor of Washington City,the as next place of meeting. On motion of Dr. Johnson, of Va., the report appointing Richmond, Va., as the next place of meeting, was adopted, and the nominations of the Committees of Arrangement and Publication were confirmed.

Dr. Hooker, chairman of the Committee of Medical Education, completed the reading of the report of the committee, concluding it with the following resolutions:

Resolved, That the abuses which exist in the modes of medical education pursued in this country demand the serious consideration of the profession.

Resolved, That free discussion in relation to the causes is an important means of effecting their removal.

Resolved, That in the opinion of this Association, no effort to remove these abuses can succeed that is not based upon a reform in the the public sentiment both of the profession and of the community.

Resolved, That this reform, so far as the profession is concerned, is to be effected mainly through its organization, and that it is, therefore, incumbent upon every physician to do all that he can to give them character and efficiency.

Resolved, That this Association have confidence in all proper efforts which have for their object a reform in the sentiment and practice of the community, in relation to medicine and the medical profession.

Resolved, That the recommendations of this Association, at its former meetings, in regard to education, both preliminary and medical, be affirmed, and that both the schools and private preceptors be still urged so to do their duty as to secure to the community a well-educated profession.

Resolved, That in the work of medical reform, while all precipitate movements should be avoided, we should aim at a steady advance, from year to year, till a thorough system of education be established by the profession throughout our country.

Dr. G. B. Wood, of Pa., asked and obtained leave to suspend the order usually observed in reports, in order to present the report of the Committee on Special Committees.

Dr. G. B. Wood, of Pa.; chairman of the committee, made the following report:

The committee to whom was referred the subject of arranging a plan of committees for future action, in place of the standing committees abolished by the Association, have the honor to report as follows:

It appears to them that the most feasible plan of accomplishing the objects of the Association, is to select certain subjects, which may be considered as suitable for investigation, and to refer these subjects to special committees, to be appointed before the close of the present session, and to report to the next. Such a selection the committee have accordingly made, and will offer to the consideration of the Association.

As an additional means of securing valuable contributions, they propose also the appointment of a committee, whose business it shall be, in the intervals between this and the next session, to receive original volunteer papers upon any subject which their authors may choose, to decide upon the merits of these papers, and to present to the Association at its next session, such of them as they may deem worthy of receiving this distinction. With a view to increase competition, they think it advisable that a prize fifty dollars, or a gold medal of that value, be awarded to each of the five papers presented to the Association, or any smaller numbei of them which the committee may consider the most meritorious, and the Association may resolve to publish.

In reference to the resolution presented in the report of the standing Committee on Medical Literature, and referred to present committee, they have only to observe that, as its ends will probably be most effectively obtained by the adoption of the general plan which they have already brought before the notice of the Association, they do not consider it expedient to make any further report.

As to the appointment of the special committee referred to, your committee think that the most convenient plan will be to refer to a special committee the nomination of a chairman for each, who shall then select, at his convenience, two individuals to aid him, with this restriction only, that the persons so selected shall be members of the Association.

To the Same Nominating Committee may be referred the appointment of the general committee, whose business will be to receive and judge of volunteer papers. As the members of this general commmittee must frequently compare opinions, it will be desirable that they should reside near each other; and it is accordingly proposed that they should be chosen from one neighborhood. If the plan be found to work well, this locality may be changed every year, so that each section of the Uniou may, in its turn, be charged with this duty. The committee would suggest that the general committee should be first chosen from members of the Association residing in Boston, or its neighborhood, as the most northern point.

To embody these suggestions in due form, the committee the following resolutions:

I. Resolved, That committees of three be appointed to investigate and report severally on the following subjects:

1st. Causes of the tubercular diathesis.

2d. Blending and conversion of the types of fever.

3d. The mutual relations of yellow fever and bilious remittent fever.

4th. Epidemic erysipelas.

5th. Acute and chronic diseases of the neck of the uterus.

6th. Dengue.

7th. The milk sickness, so called.

8tn. Endemic prevalence of tetanus.

9th. Diseases of parasitic origin.

10th. Physiological peculiarities and diseases of negroes.

11th. The action of water on lead pipes, and the diseases which proceed from it.

12th. The alkaloids which may be substituted for quinia.

13th. Permanent cure of reducible hernia.

14th. Results of surgical operations for the relief of malignant diseases.

15th. Statistics of operations for removal of stone in the bladder.

16th. Cold water dressings.

17th. The sanitary principles applicable to the construction of dwellings.

18th. The toxicological and medicinal properties of our cryptogamic plants.

10th. Agengy of the refrigeration produced through upward radiation as an exciting cause of disease.

20th. Epidemic diseases of New-England and New-York.

21st. Ditto, ditto, Pennsylvania, New Jersey, Delaware and Maryland.

22d. Ditto, ditto, Virginia and North Carolinia.

23d. Ditto, ditto, South Carolina, Georgia, Florida and Alabama.

24th. Ditto, ditto, Mississippi, Louisiana, Texas, and Arkansas.

25th. Ditto, ditto, Tennessee and Kentucky.

26th. Ditto, ditto, Missouri, Illinois, Iowa, and Wisconsin.

27th. Ditto, ditto, Indiana, Ohio, and Michigan.

II. Resolved, That a *Committee of Nomination* be appointed, whose duty it shall be to nominate one chairman for each of the above committees.

III. Resolved, That each of the chairmen thus nominated shall select, at his earliest convenience, two members of the Association to complete the committee.

IV. Resolved, That a committe of five members be appointed, to be called the *Committee for Volunteer Comunications*, whose duty it shall be, in the interval between the present and the next succeeding sessions, to receive papers upon any subject, from any persons who may choose to send them, to decide upon the merits of these papers, and to select for presentation to the Association, at its next session, such as they may deem worthy of being thus presented.

V. Resolved that the *Committee for Volunteer Communications* shall have the power to form such regulations as to the mode in which the papers are to be presented, and as to the observing of secrecy, or otherwise, as they may think proper.

VI. Resolved, That the selection of the members of this committee be referred to the same Nominating Committee, whose duty it will be to appoint the chairman of the several special committees, as above directed, with this restriction, that the individuals composing it shall reside in the same neighborhood.

VII. Resolved, That a prize of fifty dollars be awarded to each of the volunteer communications reported on favorably by the committee, and directed by the Association to be published, provided that the number to which the prize is thus awarded do not exceed five; and provided, also, if the number approved and directed to be published, exceed five, that, in such case, the prize be awarded to the five which the com-commitree may determine to be most meritorious. All of which is respectfully submitted.

GEO. B. WOOD, Chairman.

CHARLESTON, May 9th, 1851.

On motion of Dr. Huston, of Penn., the report was accepted, and the resolutions proposed by the committee were adopted.

On motion of Dr. La Roche, of Penn, the same committee were requested to make the nominations for the several special committees organized under their report.

On motion of Dr. Stevens, of New-York, it was

Resolved, That other committees may be added to those already proposed by the committes, if deemed by them advisable or necessary.

On motion of Dr. Phelps, of New-York, the report of the Committee on Medical Education was accepted, and referred to the Committee on Medical Education, and the resolutions appended to the report concurred in by the Association.

Dr. J. L. Atlee, of Pennsylvania, offered the following resolution, which was adopted.

Resolved, That it be recommended to the several State Medical Societies throughout the Union, to procure a republication of the Report ef the Committee on Medical Education, for general distribution among the profession.

Dr. Hays, of Pennsylvania, gave notice that at the next meeting of the Association, he should propose an amendment to the sixth article of the Constitution, line No. 9, so as to read $10, instead of $3.

Dr. Drake, of Ohio, then offered the following resolution:

Resolved, That in the opinion of this Association, the students of our schools should be required to matriculate within the first —— days after the opening of the session, and continue their attendance to the end of the term, taking with them evidence of the same, to be presented with the tickets of the professors, when they become candidates for degrees.

This resolution was seconded by Dr. W. Hooker, of Conn., discussed by Drs. Burrows, of Penn.; Hooker, of Conn.; F. A. Ramsay, of Tenn.; Gibson, of Penn.; Drake of Ohio; Huston, of Penn.; and finally adopted, the blank between the words "first" and "days" being unfilled.

The report of the Committe on Medical Sciences being the business next in order, the secretary read a letter from Dr. Bennet Dowler, of Louisiana, chairman of the committee, regretting his inability to be present at the meting of the Association, and presenting the report through Dr. Fenner.

Dr. Fenner, of Louisiana, read the outlines of the report of the Committee on Medical Seiences, and requested permission, in behalf of Dr. Dowler, to retain the same for revision and copy. On motion, the report was accepted, and referred to the Committee on Publication.

On motion of Dr. Phelps, of New-York, it was

Resolved, That, if a copy of the report of the Committee on Medical Sciences was not forwarded in ttme, the Committee on Publication be instructed not to delay the issue of the Transactions in consequence.

Dr. Mauran, of New-York, asked and obtained a suspension of the order of business, in order to present the following resolution, which was adopted:

Resolved, that the Committee on Publication be instructed to print conspicuously, at the beginning of the forthcoming volume of the "Transactions," the following disclaimer, viz: The American Medical Association, although formally accepting and publishing the Reports of the various Standing Committees, holds itself wholly irresponsible for the opinions, theories, or criticisms therein contained, except when so decided by special resolution.

Dr. Storer, of Massachusetts, then offered the following resolution, which was unanimously adopted:

Resolved, That the hearty thanks of this Association be presented to their late Secretary, Alfred Stille, M.D., for his constant unwearied, and invaluable services, since its first organization.

Dr. N. B. Ives, Chairman of the Committee on Adulterated Drugs, presented the report of the committee, which was read by the Secretary. A motion to refer to the Committee on Publication was debated by Drs. Gurthrie, of N. Y.; Gooch, of Va.; Ives, of Conn.; Davis, of N. Y.; Houston and Hays, of Penn.; and finally lost. On motion Dr. Davis, of N. Y.; the the report was accepted and laid on the table.

Dr. Gaillard, of S. C., chairman of the Committee on Hygiene, presented the report of the committee, and read an abstract of its contents. On motion of Dr. Hays, of Penn., the report was accepted, and referred to the Committee on Publication, with authority to print with it a paper now in preparation on the mortuary statistics of some of the larger cities.

Dr. Drake, of Ohio, offered the following amendments to the constitution, which were read and laid on the table under the rule:

"All "members by invitation" must be nominated in writing by five members of the Association, whose names shall be recorded on the minutes; when elected, they shall enjoy all the rights and privileges of delegates, and remain permanent members of the Association.

All permanent members shall be entitled to vote, and when they attend a meeting of the Association, their respective names, shall be registered, and each shall pay the sum required from a delegate.

The Secretary read a protest from the Iowa University against the representation of the Rush Medical College in this Association.

[This protest was made on the ground that Rush Medical College reduced the fees for tuition, as it asserted to the injury of neighboring schools.

We are informed by Prof. Davis, who was present when it was presented, that there was a pretty general and free expression of opinion that it was a matter with which the Association could have nothing to do; yet to dispose of the thing for the time, it was referred, and rests in the hands of the committee until next year.]

Dr. Jervey, of South Carolina, moved that the protest be referred to a select committee. This motion was discussed by Drs. Huston, of Penn.; Grimshaw, of Delaware; Gaillard, of S. C.; Davis, of N. Y.; Dickson, of S. C., Wood, of Penn.; Adams, of Mass.; Emerson, of Penn.; and finally withdrawn.

Dr. Wood, of Penn., then moved to refer the protest to the Committee on the Organization of the Association, appointed under Dr. Wood's Resolution. This motion was seconded by Dr. Huston, and adopted.

Dr. Wood, of Penn., read the following report of the committee appointed to nominate the chairman of the several special committees.

The committee to whom was referred the nomination of the chairman of the several special committees to report at the next session, and also the *Committee for Volunteer Communica-*

*tions*, report that they have fulfilled the object of their appointment, and offer the following list of chairmen to the committees first referred to, viz:

1. Dr. D. F. Condie, of Philadelphia, chairman to the Committee on the Causes of the Tubercular Diathesis.

2. S. H. Dickson, of Charleston, S. C., on the Blending and Conversion of the Types of Fever.

3. James Jones, of New Orleans, on the Mutual Relations of Yellow and Billious Remittent Fever.

4. J. B. Johnson, of St. Louis, Missouri, on Epidemic Erysipelas.

5. Charles D. Meigs, of Philadelphia, Acute and Chronic Diseases of the Neck of the Uterus.

6. J. P. Jervey, of Charleston, S. C., on Dengue.

7. Daniel Drake, of Cincinnati, Milk Sickness, so called.

8. Lopez, Mobile, Alabama, Endemic, Prevalence of Tetanus.

9. Geo. B. Wood, of Philadelphia, on Diseases of Parasitic Origin.

10. R. D. Arnold, Savannah, Georgia, on the Physiological Peculiarities and diseases of Negroes.

11. Horatio Adams, of Waltham, Mass, on the Action of Water on Lead Pipes, and the diseases which proceed from it.

12. Joseph Carson, Philadelphia, on the Alkaloids which may be substituted for Quinia.

13. George Hayward, Boston, Mass., on the Permanent Cure of Reducible Hernia.

14. S. D. Gross, Louisville, Ky., on Results of Surgical operations for the relief of malignant diseases.

15. J. R. Wood, New York, Statistics of the operation for the removal of Stone in the Bladder.

15. Charles A. Pope, St. Louis, Missouri, Water, its Topical uses in Surgery.

17. Alexander H. Stevens, New York, Sanitary principles in the coustruction of dwellings.

18. Porcher, Charleston, S. C., Toxicological and Medicinal properties of cryptogamic plants.

19. G. Emerson, Philadelphia, Agency of the refrigeration produced through upward radiation of Heat as an exciting cause of disease.

20. W. Hooker, Conn., on the Epidemics of New England and New-York.

21. J. L. Atlee, Lancaster, Penn., on the Epidemics of New Jersey, Pennsylvania, Delaware, and Maryland.

22. R. W. Haxall, Richmond, Va., on the Epidemics of Virginia and North Carolina.

23. Wm. M. Boling, Montgomery, Ala., on the Epidemics of South Carolina, Georgia, Florida, and Alabama.

24. E. H. Barton, La., on the Epidemics of Mississippi, Louisiana, Texas, and Arkansas.

25. Sutton, Georgetown, Ky., on the Epidemics of Kentucky and Tennessee.

26. Thos. Reyburn, Missouri, on the Epidemics of Missouri, Illinois, Iowa, and Wisconsin.

27. George Mendenhall, Ohio, on the Epidemics of Ohio, Indiana, and Michigan.

The following gentlemen were appointed a *Committee for Volunteer Communications*, viz: Drs. George Hayward, J. B. S. Jackson, D. H. Storer, and Jacob Bigelow, of Boston; and Dr. Usher Parsons, of Providence, R. I.

Signed on behalf of the Committee.

GEORGE B. WOOD, Chairman.

CHARLESTON, Friday, May 9th, 1851.

On motion, the report was accepted, and the nominations confirmed.

The president read an invitation from Committee of Reception of the South Carolina Medical Association to the delegates, to a steamboat excursion on the Cooper and Ashley rivers, after the adjournment of the Association.

Dr. McIntyre, of New York, moved that this Association recommend to the several State Societies the republication of the Code of Ethics and Constitution of this Association for distribution among their members; the motion was adopted.

Dr. Grimshaw, of Delaware, offered the following resolution:

Resolved, That Medical Colleges, in publishing statements of the number of Medical and Surgical cases treated at their dispensaries, act contrary to the spirit of the Code of Ethics adopted by this body. The resolution was seconded by Dr. Wood, of N. Y., debated, and finally laid on the table for future consideration.

The Association then adjourned to 5 o'clock P. M.

*Afternoon Session.*

The Vice-President, Dr. Welford, in the chair.

The special order for the afternoon session was called up, and Dr. Davis, of Illinois, read his paper entitled "An Experimental Inquiry concerning some points connected with the Process of Assimilation and Nutrition." On motion of Dr. Grimshaw, the thanks of the Association were presented to Dr. Davis for the paper just presented by him.

Dr. F.A. Ramsey, of Tenn., offered the following resolution:

Resolved, That the professors of the medical colleges be recommended to require all candidates for graduation to attend at lest two full courses of lectures. This resolution was seconded, debated, and finally rejected.

Dr. Grimshaw, of Delaware, called up as unfinished business, the resolution offered yesterday by Dr. Jones, of N. C., and not then acted upon, to which he offered the following amendment: "And that the first convention be held before the first of May, 1852." The question being taken on the resolution and the amendment, they were both laid on the table.

Dr. Phelps, of N. Y., offered the following amendment to the constitution, which, under the rule, lies over to the next meeting of the Association: "In article 2, p. 60, in the last line but one from the botom of the page, insert between the words 'endeavors' and 'to carry,' 'in reliance on divine guidance and support.' The passage will then read thus, 'and use our endeavors, in reliance on divine guidance and support, to carry into effect,' etc., etc."

Dr. Phelps, of N. Y., offered the following resolutions, which were adopted Unanimously.

Resolved, That the warmest thanks of the Association be tendered to the trustees of St. Andrew's Society for the gratuitous use of their very convenient and eligible hall, and to all those other institutions and reading-rooms which have been so freely thrown open to the inspection and use of the delegates.

Resolved, That the Committee of Reception and Arrangements receive our most grateful acknowlegments for the very handsome manner in which they have provided for the reception and entertainment of the delegates from abroad during their sojourn in the city of Charleston.

Resolved, That not only the medical profession, but the citizens generally, by private and public hospitality, have united in the manifestations of that urbanity of manner, and that unwearied and kind attention, which commands not only our profound admiration, but will be followed by the most pleasing recollections so long as life and thought shall endure.

On motion of Dr. Stevens, of N. Y., the above resolutions, and those of a similar purport adopted at the morning session, were ordered to be published in the Charleston papers.

On motion of Dr. Johnson, of Missouri, the Association adjourned *sine die.*

The Vice-President, Dr. Welford, of Va., then congratulated the Association on the happy termination of its labors, and declared the meeting adjourned.—*Medical News.*

